# Synergistic Antioxidant Activity of *Lycium barbarum* Polysaccharide and Chlorogenic Acid and Its Effect on Inflammatory Response of NR8383 Cells

**DOI:** 10.3390/foods13223696

**Published:** 2024-11-20

**Authors:** Junye Yin, Dandan Zhao, Jian Song, Ran Gao, Xuan Wang, Huan Rao, Xiaoguang Gao, Jianxiong Hao

**Affiliations:** 1 College of Bioscience & Bioengineering, Hebei University of Science & Technology, Shijiazhuang 050018, China; 15630195857@163.com (J.Y.); seven_ran0207@163.com (R.G.); bible066@163.com (X.W.); raohuan881210@163.com (H.R.); haojianx@hebust.edu.cn (J.H.); gaoxiaoguang23@hotmail.com (X.G.); 2 Shandong Center for Disease Control and Prevention, Jinan 250014, China; songirlike@163.com; 3 Hebei Province Functional Food Technology Innovation Center, Shijiazhuang 050018, China

**Keywords:** polysaccharides and polyphenols, synergistic effect, cell inflammatory injury, antioxidant and anti-inflammatory

## Abstract

It is inevitable for polyphenols and polysaccharides to interact during food preparation. Modifications in microstructure can lead to changes in the physical and chemical properties of food systems, which in turn may influence the nutritional characteristics and functional activities of the food. Recent studies have shown that, in addition to traditional Chinese medicine compounds, certain natural polysaccharides and polyphenols exhibit significant anti-inflammatory and antioxidant properties. These compounds are also associated with beneficial therapeutic effects for the prevention and treatment of acute lung injury. The objective of this study was to examine the synergistic antioxidant effects of chlorogenic acid (CA) and *Lycium barbarum* polysaccharide (LBP) in various ratios, along with their combined antioxidant and anti-inflammatory effects on LPS-induced inflammation in rat alveolar macrophages. Using the Combination Index (CI), which quantifies the synergistic or antagonistic effect of two substances, all four combinations showed synergistic antioxidant properties over a range of concentrations by in vitro antioxidant property experiments. However, based on comparing them, the four group ratios exhibited the highest antioxidant activity of the infusion at CA:LBP = 1:7, indicating synergistic interactions (CI < 1). In addition, the antioxidant and anti-inflammatory effects of the CA-LBP complex were observed to alleviate cellular inflammatory injury by reducing LPS-induced nitric oxide and reactive oxygen species production and inhibiting the release of inflammatory factors such as TNF-α and IL-6.

## 1. Introduction

Acute lung injury (ALI) is an acute inflammatory condition affecting the lungs, resulting from various direct or indirect factors (e.g., sepsis, pneumonia, alveolar hemorrhage, toxic gases). These factors cause damage to both alveolar epithelial cells and capillary endothelial cells, leading to diffuse interstitial and alveolar edema. This condition presents with adverse manifestations including hypoxemia and respiratory distress. In severe cases, it can progress to acute hypoxic respiratory insufficiency, posing a significant threat to the patient’s life [1]. Damage to the alveolar epithelium and lung endothelium is a critical pathophysiological characteristic of acute respiratory distress syndrome (ARDS). Inflammatory injuries cause the increased permeability of the alveolar capillaries, leading to the accumulation of protein-rich fluid in the alveolar space. Previous research has demonstrated that alveolar macrophages are essential immune cells within lung tissue, equipped with strong phagocytic and immune capabilities. These macrophages emit a variety of pro- and anti-inflammatory cytokines when the lungs are infected by pathogens, and these cytokines are essential for the host’s defensive systems in healthy lungs [2]. Furthermore, these cells not only help control lung injury by removing pathogens and infected or apoptotic cells through phagocytosis and intracellular killing but also maintain airway immune homeostasis [3]. Despite extensive studies on the pathogenesis of acute lung injury (ALI) in recent years, effective therapeutic measures or specific drugs remain lacking.

*Lycium barbarum*, often known as goji berry, has been important in traditional Chinese medicine for more than 2000 years. The fruit’s primary active element, *Lycium barbarum polysaccharide* (LBP), accounts for 5–8% of its dried form [4]. LBP has a variety of biopharmacological effects, including anti-inflammatory [5], antioxidant [6], hypoglycemic [7], and anti-cancer ones [8], and intestinal microbiota regulation [9]. The biological activities of LBP have been shown to help minimize lung inflammation and injury. Animal studies have revealed that LBP promotes Adenosine 5′-monophosphate (AMP)-activated protein kinase (AMPK) activity and decreases lung inflammation and edema in a hyperoxic ALI model [10]. Currently, the research shows that LBP protects asthmatic mice by reducing airway inflammation, decreasing pulmonary epithelium oligosaccharide glycation, and regulating intestinal microbiota dysregulation [11].

Polyphenolic antioxidant chlorogenic acid (CA) is commonly found in human diets, with coffee, fruits, and vegetables being the primary sources. Other sources include apples, pears, and berries. CA exhibits numerous pharmacological properties, including antioxidant [12,13], anti-inflammatory [14], antibacterial [15], neuroprotective [16], and anticancer activities [17], as well as hepatoprotective effects [18]. Research has shown that CA can effectively treat colitis caused by dextran sulfate sodium salt (DSS) by activating the Nrf2/HO-1 pathway, which decreases oxidative stress and inflammatory reactions [3]. CA also promotes the intestinal barrier and has a protective effect on ARDS by reducing bacterial loads in the lungs and attenuating inflammatory injuries. This is achieved by upregulating GPR37 expression and enhancing the phagocytosis of alveolar macrophages [19]. Additionally, CA binds to membrane-bound protein A2 to prevent tumor cell migration and proliferation. According to the studies, this results in a decrease in the production of anti-apoptotic genes downstream of NF-κB, which in turn reduces the proliferation of A549 cells both in vitro and in vivo [20].

Many natural active substances found in functional meals can have either an antagonistic or synergistic effect when combined. Compared to a single active component, this may result in either increased or decreased biological activity. The relationship between two bioactive compounds can be classified as additive, synergistic, or antagonistic. Synergism occurs when two bioactive compounds produce similar effects within the body and can even create additional effects when used together. The synergistic activity of two compounds is usually measured using an isobologram, while the Combination Index (CI) is another standard measurement based on the dose–effect relationship of both compounds. A CI value of less than 1 indicates synergy, a CI of 1 indicates addition, and a CI greater than 1 indicates antagonism. Polyphenols and polysaccharides are the main active components of plants, and both can coexist in covalent and non-covalent forms [21]. Studies have shown that the interactions between polyphenols and polysaccharides are mainly non-covalent and covalent interactions. Among them, non-covalent interactions include the adsorption of natural and oxidized polyphenols to the cell wall matrix, mostly due to hydrogen bonding and hydrophobic interactions between ions. During processing, polyphenols and polysaccharides can spontaneously and rapidly undergo non-covalent binding. It has been found that the non-covalent combination of the two can affect the stability, sensory quality and nutritional properties of food products [22,23]. Covalent interaction is due to the intermolecular covalent bond between polyphenols and polysaccharides, forming covalent complexes, which are mostly induced by free radicals, enzymatic reaction, and carbodiamine crosslinking, similar to the oxidation mechanism of o-quinone. Most polyphenols and polysaccharides produce hydrophobic interactions by aggregating together, at which point intermolecular forces dominated by hydrophobic interactions are aided by hydrogen bonding to enhance their binding. It was found that in the aqueous mixture system, lotus root polysaccharide and polyphenol can spontaneously combine through non-covalent interaction. Under the condition of a 1:4 mass ratio of lotus root polysaccharide/chlorogenic acid, the CA binding amount is 2179.09 mg/g [24,25].

More recent research has demonstrated that polyphenol–polysaccharide complexes have anti-inflammatory [26], hypoglycemic [27], synergistic antioxidant [28], intestinal health-promoting [29,30], and biological activity-inhibiting effects on cancer cell proliferation [31], as demonstrated by a decrease in the pulmonary index, a more complete lung morphology, and a decrease in inflammatory cells and mediators; for instance, the combination of total flavonoids (HCFs) and polysaccharides (HCPs) from Houttuynia cordata considerably improved viral pneumonia [32]. Hepatocellular cancer cells were synergistically inhibited by high-molecular-weight oolong tea polysaccharides (OTPS1s) and polyphenols (OTPs), and mice’s immunological and antioxidant capacities were markedly enhanced following co-administration [33].

The purpose of this study was to look into the synergistic antioxidant activity of chlorogenic acid and *Lycium barbarum* polysaccharide in varied amounts. We analyzed the efficacy of this combination in scavenging ABTS free radicals, DPPH free radicals, and hydroxyl free radicals. The assessment of the synergistic antioxidant activity was carried out using the Chou–Talalay principle. Additionally, we explored the combined antioxidant and anti-inflammatory effects of the CA and LBP complex on LPS-induced inflammation in rat alveolar macrophages. This investigation included evaluating the cells’ capacity to manage oxidative stress and regulate pro-inflammatory cytokines, and discussing the potential mechanisms underlying the observed synergy.

## 2. Materials and Methods

### 2.1. Reagents and Standards

*Lycium barbarum* polysaccharide extract was purchased from Beijing Solaibao Technology Co., Ltd. (Beijing, China). Chlorogenic acid was sourced from Shanghai Aladdin Biochemical Technology Co., Ltd. (Shanghai, China). PMP was obtained from Shanghai Titan Technology Co., Ltd. (Shanghai, China). The following analytically pure substances were purchased from Shanghai Pure One Biotechnology (Shanghai, China): potassium persulfate, hydrogen peroxide, DPPH, ABTS, and salicylic acid. Mannose, rhamnose, glucose, arabinose, galactose, and galacturonic acid (reference standard, purity ≥98% each) were purchased from Sigma-Aldrich (Shanghai, China). Rat alveolar macrophage NR8383 was purchased from Pricella Biological Company (Wuhan, China). Serum-free cell-freezing solution and lipopolysaccharide (LPS) were purchased from Solarbio Life Science (Beijing, China). PBS, penicillin-streptomycin solution (100×), 0.25% trypsin, reactive oxygen species assay kits, and the CCK-8 reagent were acquired from Meilun Biotechnology Co. Ltd. (Dalian, China). Nanjing Jiancheng Biotechnology Co., Ltd. (Nanjing, China) was the supplier of the NO kit. We bought the rat tumor necrosis factor α (TNF-α) and rat interleukin 6 (IL-6) ELISA kits from Wuhan Elabscience Biotechnology Co., Ltd. (Wuhan, China).

### 2.2. Determination of Polysaccharide Content

The phenol–sulfuric acid technique was used to measure the amount of *Lycium barbarum* polysaccharide [34]. First, 1 milliliter of each 5% phenol solution was combined with LBP. Following that, 5 mL of sulfuric acid was added gradually and allowed to react for 30 min in boiling water. After cooling, the solution’s absorbance was measured at 490 nm. The polysaccharide content was determined using glucose and is given as milligrams of glucose equivalent (GE) per 100 milliliters of LBP injection.

### 2.3. Monosaccharide Composition Analysis

The composition of monosaccharides was studied by high-performance liquid chromatogram–PMP pre-column purification [35]. To produce the sample, a polysaccharide sample (10 mg) was hydrolyzed with 5 mL 2 mol/L trifluoroacetic acid (TFA) at 90 °C for 3 h. After hydrolysis, it was dried under nitrogen (n_2_), and 1 mL of 50% ethanol was added. Next, 200 µL of PMP–methanol solution (0.5 mol/L) and NaOH solution (0.3 mol/L) were mixed to form a mixed standard solution. The mixture was incubated in a 70 °C water bath for 30 min. Then, chloroform was added to extract the organic phase for a total of three times. The resulting solution was filtered on a 0.45 µm membrane prior to HPLC analysis. Under the following circumstances, the resulting samples were subjected to HPLC analysis: 3.5 μm, 150 mm × 4.60 mm Symmetry^®^C18 column. Acetonitrile and 0.05 mol/mL phosphate buffer (pH 6.8) at a flow rate of 0.6 mL/min made up the mobile phase; 40 °C was the column temperature, while 250 nm was the ultraviolet detection wavelength.

### 2.4. Deproteinization

The Sevag reagent method is based on the idea that organic solvents like chloroform denature proteins. To extract the protein from polysaccharides using the Sevag reagent method, dissolve the required amount of polysaccharide powder and transfer it to a dispensing funnel. Add Sevag reagent (chloroform/n-butanol volume ratio: 4:1). The volume ratio of the polysaccharide solution to Sevag’s reagent was approximately 3:1, and the mixed solution was well shaken and allowed to stratify before the aqueous phase was retained, and the procedure was performed three times [36].

### 2.5. ABTS Radical Scavenging Ability

A total of 2.6 mmol/L K_2_S_2_O_8_ and 7.3 mmol/L ABTS^+^ solution was mixed and then left in the dark for the ABTS test [37]. By diluting the ABTS^+^ solution with 80% methanol until the absorbance was 0.7–0.8 (at 734 nm), the ABTS^+^ working solution was produced. Finally, 200 μL of sample extract was mixed with 2 mL of ABTS^+^ working solution, and the absorbance at 734 nm was measured six minutes later. This is how the clearance was determined:

(1) $$\mathrm{Scavenging}\;\mathrm{ability}\;(\%)=\left(1-\frac{A_{1}-A_{2}}{A_{0}}\right)\times 100\%$$

### 2.6. DPPH Radical Scavenging Ability

The DPPH radical scavenging activities of the samples were assessed following the method outlined by Muruthi et al. [38]. The DPPH working solution was made by dissolving 0.004 g of DPPH in 100 mL of 80% methanol, yielding a concentration of 0.01 mmol/L. Following that, 200 μL samples at various concentrations were mixed with 3.5 mL of the DPPH working solution, and the combination was left to incubate for 30 min at room temperature, away from direct sunlight. Finally, the absorbance of the combination at 517 nm was measured using a spectrophotometer. Equation (2) was used to determine the sample’s rate of DPPH free radical elimination.

(2) $$\mathrm{Scavenging}\;\mathrm{ability}\;(\%)=\left(1-\frac{A_{1}-A_{2}}{A_{0}}\right)\times 100\%$$

### 2.7. Hydroxyl Radical Scavenging Activity

When H_2_O_2_ and FeSO_4_ are combined, -OH is created. Salicylic acid is then added to the -OH to create 2, 3-dihydroxybenzoic acid, which has a unique absorbance at 510 nm. Adding antioxidants that can eliminate hydroxyl radicals to the aforementioned chemical system can decrease the amount of colorful compounds that develop and the system’s absorbance value [39]. After mixing the samples with 6 mmol/L FeSO_4_ solution and 6 mmol/L H_2_O_2_ solution (two milliliters each), they were allowed to sit at room temperature for ten minutes. After 30 min, the absorbance value was measured at 510 nm using the same volume of 6 mmol/L salicylic acid solution. Instead of sample solution, sample solvent was utilized in the blank group. Equation (3) was used to determine the control group’s hydroxyl radical clearance rate, and pure water was used in its place.

(3) $$\mathrm{Scavenging}\;\mathrm{ability}\;(\%)=\left(1-\frac{A_{1}-A_{2}}{A_{0}}\right)\times 100\%$$

### 2.8. Complex Combination of LBP and CA

The free radical scavenging rates of *Lycium barbarum* polysaccharide and chlorogenic acid were evaluated as individual components. The IC_50_ values for the antioxidant activity of chlorogenic acid are relatively low, meaning that even small concentrations of these compounds can demonstrate significant scavenging capability. In contrast, *Lycium barbarum* polysaccharides are usually found in higher concentrations, but they often require larger quantities to achieve a similar level of scavenging effect. Consequently, combining *Lycium barbarum* polysaccharides with lower concentrations of chlorogenic acid can effectively enhance their overall efficacy. The compound of different proportions was prepared by the physical mixing of *Lycium barbarum* polysaccharide solution and chlorogenic acid solution at ratios of 1:3, 1:5, 1:7 and 1:9. In addition, six different concentration levels were established for each ratio, and the scavenging capacity of each combination against DPPH, ABTS, and hydroxyl radicals was assessed. Finally, the combined drug theory of Chou–Talalay was used to determine the synergistic antioxidant effects of different proportions of complexes. All the reported data are averages of three or more independent experiments.

### 2.9. Cell Culture

The Chinese cell bank (Wuhan, China) provided the NR8383 alveolar macrophages, which were cultivated at 37 °C with 5% CO_2_ in Ham’12k medium supplemented with 20% FBS and 1% penicillin-streptomycin.

### 2.10. Cell Viability Assay

The CCK-8 technique was used to evaluate cell viability. Following their inoculation at a density of 5 × 10^4^ cells/well in 96-well plates, NR8383 macrophages were exposed to different concentrations of CA-LBP for a duration of 24 h (sample concentrations of 160, 240, 320, 400, 480, and 560 μg/mL). A 0.22 μm filter (Shanghai Mosu Scientific Instruments and Materials, Shanghai, China) was used to filter the CA-LBP samples. After that, the CCK-8 reagent was applied and incubated for one hour at 37 °C, and its degree of reduction was evaluated using an enzyme label at 450 nm [40].

### 2.11. Measurement of Nitric Oxide (NO)

NR8383 macrophages were inoculated in 24-well plates at a density of 1 × 10^5^, and the experiments were divided into control, model, and experimental groups (40 μg/mL CA, 280 μg /mL LBP, and 40 μg/mL CA + 280 μg/mL LBP). Pretreatment of the cells was carried out for 24 h, followed by stimulation of the cells with 1 μg/mL LPS for 24 h. The cell supernatant was taken from each well, following the instructions of the Nitrate Reductase Assay NO Kit. The reaction reagent was added to the sample as instructed and left for 40 min at room temperature. Then, the supernatant was centrifuged, and the colorant was added, and the reaction was left at room temperature for ten minutes, and the light absorption value at 550 nm was measured [41].

### 2.12. Reactive Oxygen Species Assay

Cellular reactive oxygen species were measured using the DCFH-DA kit. The DCFH-DA kit uses a fluorescence probe to detect reactive oxygen species. Since DCFH-DA is non-fluorescent, it can easily cross cell membranes. Once within the cell, intracellular esterases can hydrolyze it to create DCFH, which is easy to label into the cell because it does not cross the cell membrane.

NR8383 macrophages were inoculated in 24-well plates (cell density of 5 × 10^5^/mL), and the experiments were divided into a control group, a model group (induced by 1 μg/mL LPS), and an experimental group with different concentrations (40 μg/mL CA, 280 μg/mL LBP, and 40 μg/mL CA + 280 μg/mL LBP). Following a 24 h pretreatment with CA-LBP, the NR8383 cells were stimulated for an additional 24 h period with LPS (1 µg/mL). After centrifuging the cell suspension, the supernatant was disposed of. The cells were then suspended, incubated for 20 min at 37 °C in a cell incubator, and 1 mL of diluted DCFH-DA was added to the cell precipitation. Following two PBS washes, the cells were rehung. Lastly, three replicates were established in each experimental group, and the absorbance of the cells in each group was assessed using a fluorescent enzyme marker [42].

### 2.13. ELISA Assessment

Following a 24 h pretreatment with LBP-CA, the NR8383 cells were stimulated for another 24 h period with LPS (1 µg/mL). Samples and standards containing TNF-α protein were added separately to bind to the TNF-α monoclonal antibody and washed to remove free components. Biotinylated anti-rat TNF-α antibody and horseradish peroxidase-labeled affinity protein were added, at which point specific binding of the biotin to the affinity protein occurred, and the free components were washed away. Finally, a substrate, horseradish peroxidase, was added to convert colorless chromogens to blue, followed by a termination solution to turn them yellow, and the absorbance at 450 nm was measured.

### 2.14. Data Analysis

The data are expressed as mean ± standard error. To determine the statistical significance of the data, a one-way analysis of variance (ANOVA) was performed using SPSS 22.0 software. *p* < 0.05 was the cutoff point for statistical significance. Origin 9.0 software was used to draw the curve. In this chapter, Chou–Talalay’s CompuSyn1.0.1 program was used to assess the combination of chlorogenic acid and LBP’s capacity to scavenge hydroxyl, DPPH, and ABTS free radicals.

## 3. Results

### 3.1. Component Analysis

High-performance liquid chromatography with pre-column derivatization was used to examine the monosaccharide content in LBP. The HPLC retention durations of reference sugars were used to determine the monosaccharide composition of LBP. Mannose has a retention duration of 10.689 min, rhamnose 14.325 min, galacturonic acid 14.528 min, glucose 18.946 min, galactose 20.890 min, and arabinose 23.286 min. Six conventional monosaccharide compositions were discovered, ranging from low to high. This rule states that the mass fraction of the LBP sample was 44.7% and that the primary constituents of LBP were mannose, rhamnose, galacturonic acid, glucose, galactose, and arabinose. The results showed that the dominant monosaccharide in LBP was galactose (Table 1). This differs from previous reports on LBP from the same species [35,43], indicating structural differences due to various factors, such as different plant collection sources, distinct isolation procedures, or variations in the methods used to determine the proportions of monosaccharides. PMP-RP-HPLC is a superior technique to GC for the qualitative and quantitative analyses of constituent monosaccharides released from LBP. It can also be employed to identify diverse types of monosaccharides in LBP.

### 3.2. In Vitro Antioxidant Activity of CA and LBP

Table 2 displays the in vitro antioxidant activity test for chlorogenic acid at varying concentrations. The results demonstrate that chlorogenic acid has a maximum scavenging effect of over 80% on ABTS and DPPH within the same concentration range. It has a relatively poorer capacity to scavenge hydroxyl radicals, however. As shown in Table 3, different amounts of LBP were used to evaluate its in vitro antioxidant property. LBP’s capacity to scavenge hydroxyl, DPPH, and ABTS radicals grew as the quantity in the trial ranged from 1 to 7 mg/mL. The scavenging capacity of hydroxyl and DPPH radicals was within the same concentration range of 65%, whereas the maximal scavenging rate of ABTS was 90.28%.

The scavenging rate of hydroxyl, DPPH, and ABTS radicals was used to assess the in vitro antioxidant activity of LBP and chlorogenic acid. The results above demonstrated that both compounds exhibited good antioxidant activity. Chlorogenic acid and LBP had respective IC_50_ values of 70.53 μg/mL and 2.12 mg/mL against ABTS radicals. The ratio of chlorogenic acid and LBP used in combination was set up as 1:3, 1:5, 1:7, and 1:9 for the following experiments, using the two IC_50_ values against ABTS radicals and the pre-experiment.

### 3.3. In Vitro Antioxidant Activity of CA-LBP

#### 3.3.1. ABTS Scavenging Capacity of CA-LBP

**Figure 1:**
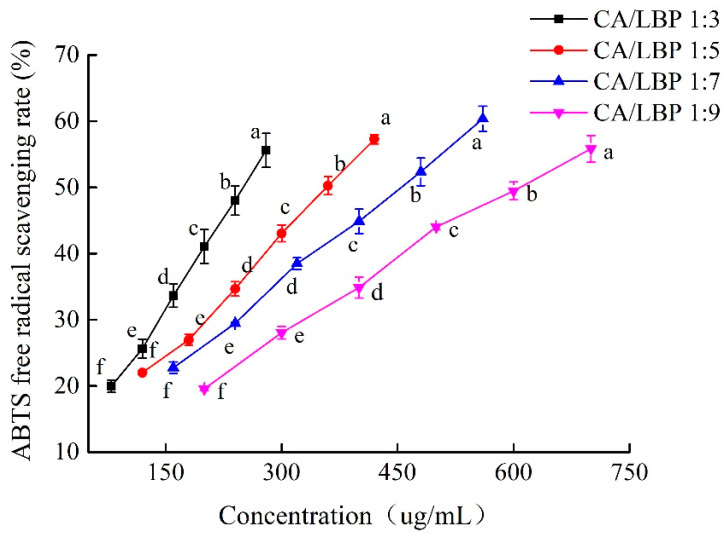
The ABTS radical scavenging activity of CA in combination with LBP. Every experiment was run in triplicate, and the means of the various lowercase letters differ significantly (*p* < 0.05). *n* = 3.

The ABTS radical scavenging activity curves illustrated in Figure 1 indicate that the scavenging ability of the four groups of chlorogenic acid–*Lycium barbarum* polysaccharide complexes (CA-LBP) increased as the concentration of ABTS radicals increased. This demonstrates a clear and effective quantitative relationship. The strongest scavenging ability for ABTS radicals was observed when the ratio of CA-LBP complexes was 1:7.

**Figure 2:**
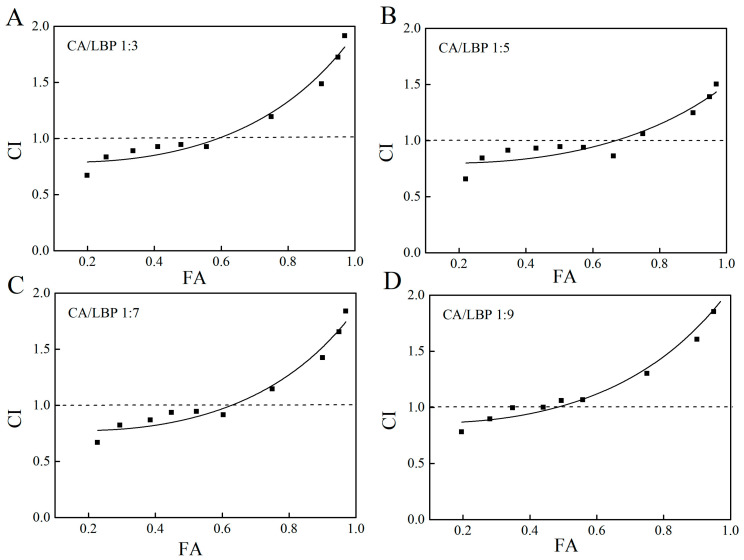
Fa-CI plot for CA in combination with LBP. (**A**) CA/LBP = 1:3 free radical scavenging ability CI value of ABTS. (**B**) CA/LBP = 1:5 free radical scavenging ability CI value of ABTS. (**C**) CA/LBP = 1:7 free radical scavenging ability CI value of ABTS. (**D**) CA/LBP = 1:9 free radical scavenging ability CI value of ABTS. (Fa represents the action fraction at the corresponding concentration. CI is the combined index, where CI > 1 indicates antagonism, CI = 1 signifies addition, and CI < 1 denotes synergy. Preparation method of the mixture: the solutions of *Lycium barbarum* polysaccharide and chlorogenic acid were combined in the following ratios: 1:3, 1:5, 1:7, and 1:9. Furthermore, six distinct concentration levels were defined for each ratio, and subsequent tests were performed to assess theirt antioxidant capacity. All reported data represent averages obtained from three or more independent experiments.

The ability of chlorogenic acid and *Lycium barbarum* polysaccharide to scavenge ABTS radicals, DPPH radicals, and hydroxyl radicals in combination was analyzed using CompuSyn1.0.1 software developed by Chou–Talalay. Chou and Talalay proposed the Combination Index (CI) in 1983 to quantify the synergistic or antagonistic effects of two substances, and a CI ≤ 1 and >1 denote synergistic, additive, and antagonistic effects, respectively. As shown in Figure 2, when the ratio of chlorogenic acid (CA) to LBP is 1:3, the fitting curve of the CI value is lower than the clearance rate of 56%, and the interaction index (CI) is less than 1. This indicates that chlorogenic acid and LBP exhibit a certain degree of synergistic antioxidant effects within this ratio. At a CA-LBP ratio of 1:5, the clearance rate of the fitting curve remains below 67%, with the CI value consistently under 1, suggesting that the combination also demonstrates some synergistic ability. When the CA-LBP ratio is 1:7, the clearance rate of the fitted curve drops to less than 60%, and the CI value remains below 1, indicating that there is a notable synergistic antioxidant effect in the concentration range of 160 μg/mL to 560 μg/mL. Lastly, at a CA-LBP ratio of 1:9, the clearance rate of the fitting curve falls below 45%, with the CI value also less than 1, which suggests that the combination possesses a certain level of synergistic antioxidant capacity in the lower concentration range.

#### 3.3.2. DPPH Scavenging Capacity of CA-LBP

**Figure 3:**
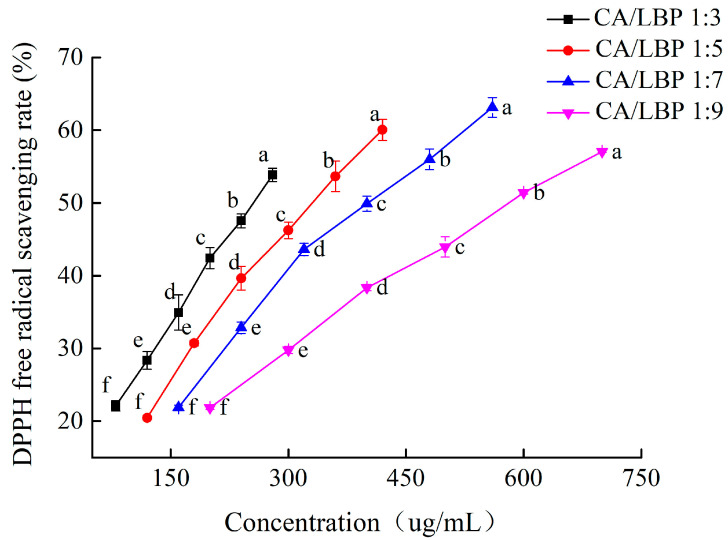
The DPPH radical scavenging activity of CA in combination with LBP. All experiments were performed in triplicate and means with different lowercase letters are significantly different (*p* < 0.05), *n* = 3.

The DPPH free radical scavenging activity curve, depicted in Figure 3, indicates that the scavenging ability of four different proportions of the CA-LBP complex on DPPH free radicals improves with increasing concentrations. Notably, the strongest scavenging ability occurs when the CA-LBP complex ratio is 1:7.

**Figure 4:**
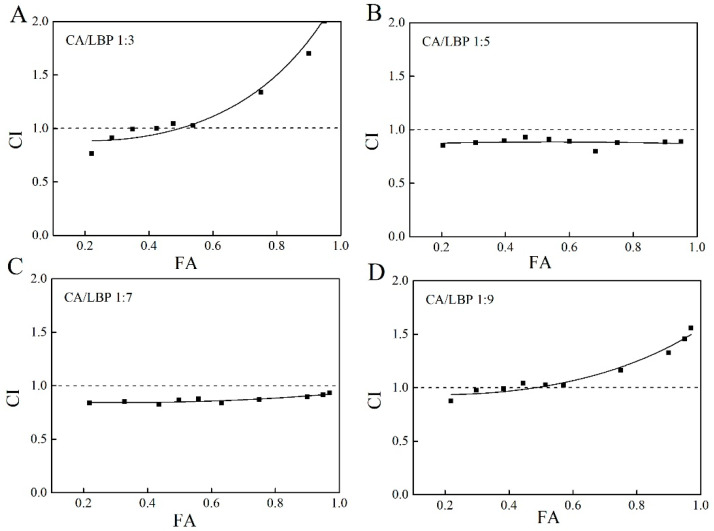
Fa-CI plot for CA in combination with LBP. (**A**) CA/LBP = 1:3 free radical scavenging ability CI value of DPPH. (**B**) CA/LBP = 1:5 free radical scavenging ability CI value of DPPH. (**C**) CA/LBP = 1:7 free radical scavenging ability CI value of DPPH. (**D**) CA/LBP = 1:9 free radical scavenging ability CI value of DPPH.

Figure 4A presents the DPPH free base associative digital map, with a CA-LBP ratio of 1:3. The clearing rate of the fitted curve is below 42%, and the interaction index (CI) is less than 1. This suggests that the CA-LBP complex demonstrates a notable cooperative antioxidant function at lower concentration levels. In Figure 4B,C, the results for CA-LBP ratios of 1:5 and 1:7 are displayed. In both scenarios, the clearance rates of the fitted curves exceed 97%, and the CI values remain below 1, suggesting that the combination of chlorogenic acid and LBP exhibits synergistic antioxidant properties at these ratios. At a ratio of 1:9, the clearance rate of the fitting curve declines to below 40%, while the CI value continues to be under 1, indicating that the complex retains some synergistic antioxidant capacity.

#### 3.3.3. Hydroxyl Radical Scavenging Ability of CA-LBP

**Figure 5:**
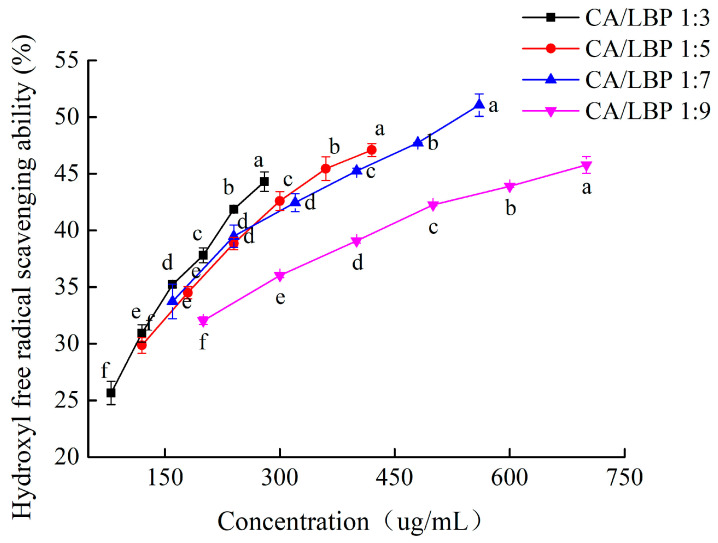
The hydroxyl radical scavenging activity of CA combined with LBP. All experiments were performed in triplicate and means with different lowercase letters are significantly different (*p* < 0.05), *n* = 3.

As shown in Figure 5, the curve depicting hydroxyl radical scavenging activity shows that the scavenging ability of the CA-LBP complex in four distinct proportions increases with the concentration. At a ratio of 1:7 for the CA-LBP complex, its scavenging ability is the strongest.

**Figure 6:**
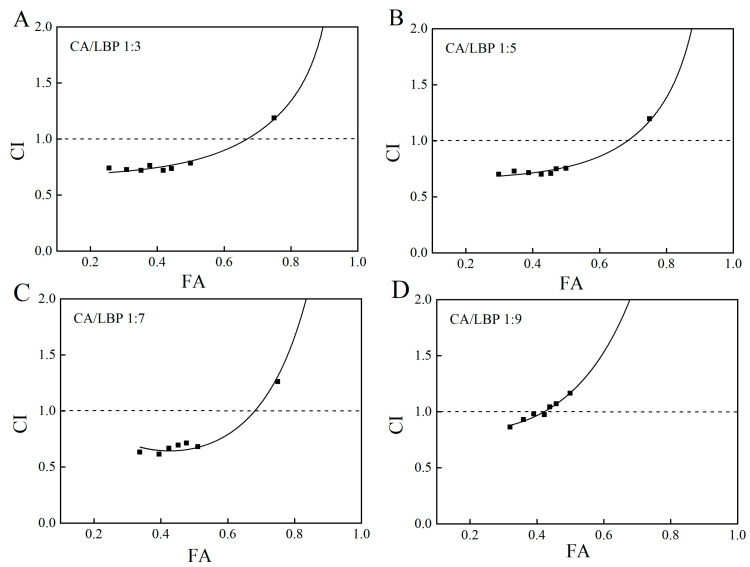
Fa-CI plot for CA in combination with LBP. (**A**) CA/LBP = 1:3 CI for hydroxyl radical scavenging capacity. (**B**) CA/LBP = 1:5 CI for hydroxyl radical scavenging capacity. (**C**) CA/LBP = 1:7 CI for hydroxyl radical scavenging capacity. (**D**) CA/LBP = 1:9 CI for hydroxyl radical scavenging capacity.

The hydroxyl radical association index diagrams for CA-LBP ratios of 1:3 and 1:5 are depicted in Figure 6A,B. The fitting curves indicate a range of 12% to 65%, with CIs all below 1. This suggests that the CA-LBP complex exhibits synergistic antioxidant effects within this concentration range. At a ratio of 1:7, the clearance rate of the fitted curve varies between 5% and 65%, and the CI remains below 1, indicating that CA-LBP also possesses synergistic antioxidant properties. However, at a CA-LBP ratio of 1:9, the clearance rate of the fitting curve drops to less than 42%, with the CI still under 1, suggesting that the complex retains some synergistic antioxidant capacity at lower concentrations.

### 3.4. Effects of Cell Activity

**Figure 7:**
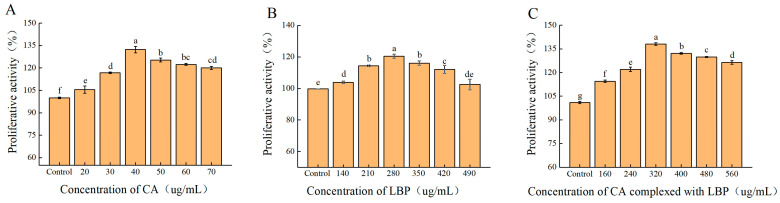
Effects of CA, LBP, and their complexes on cell viability. (**A**–**C**) Results of the CCK-8 assay in NR8383 murine macrophages of CA, LBP, and CA complex with LBP for 24 h, respectively. All experiments were performed in triplicate and means with different lowercase letters are significantly different (*p* < 0.05), *n* = 3.

Following a 24 h administration of varying quantities of *Lycium Barbarum* polysaccharides (LBPs), chlorogenic acid (CA), and their complexes, we assessed cell viability using the CCK-8 test. Our results showed that at concentrations ranging from 20 μg/mL to 70 μg/mL, CA promoted cell proliferation without any toxic effects. The rate of cell proliferation was faster between 20 μg/mL and 40 μg/mL and then decreased (Figure 7A). Similarly, LBP promoted cell proliferation without any toxic effects at concentrations ranging from 140 μg/mL to 490 μg/mL. The rate of cell proliferation was faster between 140 μg/mL and 280 μg/mL (Figure 7B). Both CA and LBP complexes promoted cell proliferation at concentrations ranging from 160 μg/mL to 560 μg/mL. Therefore, we conducted follow-up experiments using 40 μg/mL of CA, 280 μg/mL of LBP, and 320 μg/mL of CA-LBP complex (Figure 7C).

### 3.5. Effect of CA-LBP on LPS-Induced Inflammatory Factor Release from NR8383 Cells Detected by ELISA

**Figure 8:**
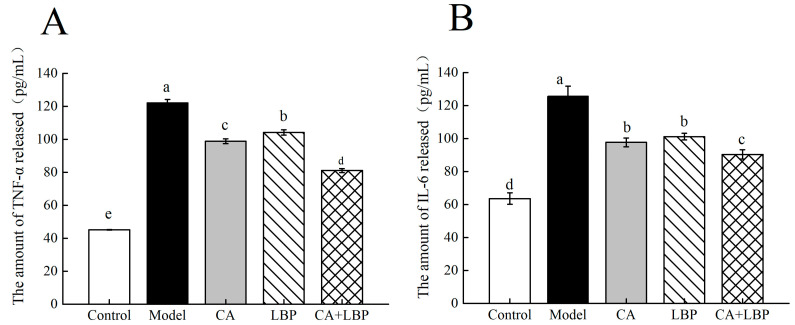
LPS induces TNF-α and IL-6 release from NR8383 cells. (**A**,**B**) Levels of TNF-α and IL-6 in culture supernatant as detected by ELISA, respectively. All experiments were performed in triplicate and means with different lowercase letters are significantly different (*p* < 0.05), *n* = 3.

Gram-negative bacteria’s outer cell wall contains LPS, sometimes referred to as endotoxin. This substance is a major contributor to inflammatory damage in the body and can trigger macrophages to release pro-inflammatory cytokines like TNF-α and IL-6. Following a 24 h treatment with 1 μg/mL LPS, all groups’ levels of cellular inflammatory components significantly increased in comparison to the control group. Compared with the model group, LBP, chlorogenic acid, and the complex of the two inhibited the release of the cellular inflammatory factors. The alleviation of cellular inflammatory injury by the CA-LBP complex was a little bit more significant, and the release of TNF-α and IL-6 was 81.1 pg/mL and 90.4 pg/mL, respectively (Figure 8).

### 3.6. Inhibitory Effect of CA-LBP on LPS-Induced NO Release

**Figure 9:**
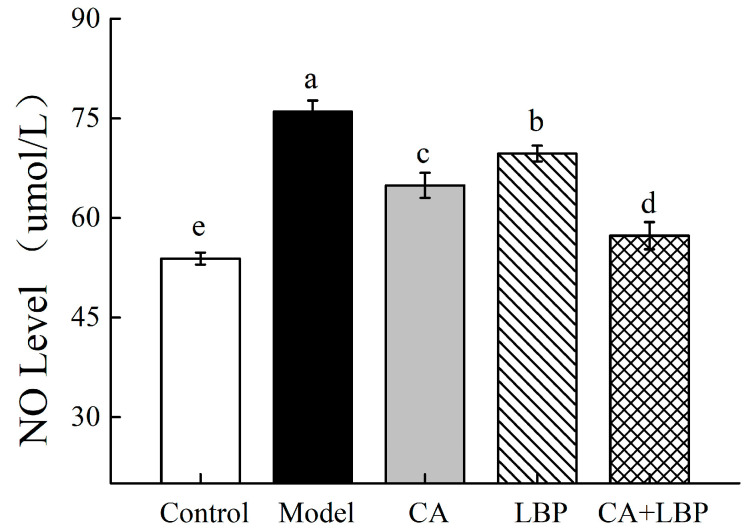
Effect of LPS-induced NO release from NR8383 cells. All experiments were performed in triplicate and means with different lowercase letters are significantly different (*p* < 0.05), *n* = 3.

In vivo, NO plays a variety of physiological roles, but excessive NO release causes negative cellular effects, such as oxidative damage to cells. Compared to the model group, NO release in the control group was much higher, showing that a cellular inflammatory model had been successfully established (Figure 9). While the CA-LBP complex significantly inhibited LPS-induced NO production, the NO release in the CA (40 μg/mL), LBP (280 μg/mL), and CA + LBP (40 + 280 μg/mL) concentration groups was significantly lower than that in the LPS group, indicating that both the single component and the complexes were able to reduce the release of NO from the inflammatory macrophage cells.

### 3.7. Effect of CA-LBP on LPS-Induced ROS Release in NR8383 Cells

**Figure 10:**
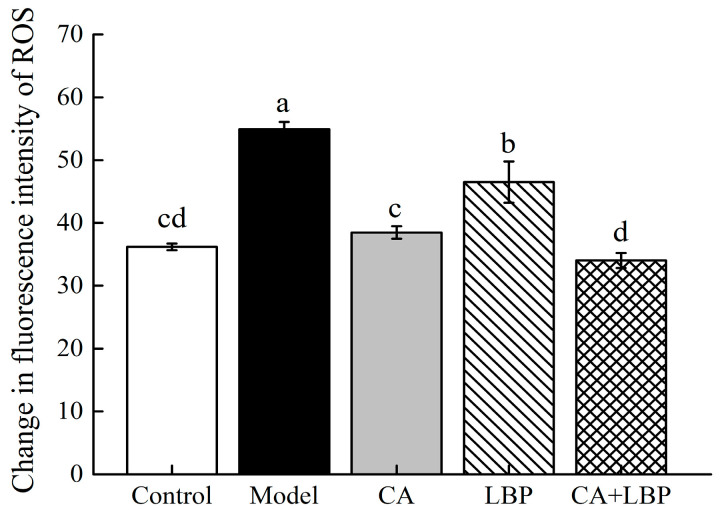
LPS induces ROS release from NR8383 cells. All experiments were performed in triplicate and means with different lowercase letters are significantly different (*p* < 0.05), *n* = 3.

ROS is the most intuitively observed indicator after cells are subjected to oxidative damage, and ROS release was detected by employing a DCFH-DA fluorescent probe and utilizing a fluorescent zymography instrument (Figure 10). LPS induced a significant increase in the release of ROS in the NR8383 cell model group when compared with that of the blank control group, which indicated that a certain concentration of CA and LBP, as well as the complexes of the two, could effectively reduce the release of ROS induced by LPS.

## 4. Discussion

ALI is described as changes in lung tissue that include epithelial malfunction, excessive inflammation, and pulmonary edema [44]. The etiology of ALI is uncertain; however, it is thought to entail lung epithelial and endothelial damage, pro-inflammatory modulator synthesis, severe oxidative stress, and a massive influx of neutrophils into the lungs. Inflammatory reactions can stimulate the overproduction of ROS, while oxidative stress can also drive inflammatory responses. Oxidative stress and strong inflammatory responses are the two main causes of ALI [45]. Furthermore, endotoxin can drive excessive cytokine, chemokine, and ROS production. Pro-inflammatory mediators produced by LPS-stimulated macrophages play an important role in beginning and perpetuating inflammation [46]. It was discovered that CA boosted the phagocytosis of the cells and markedly improved the inflammatory response of lipopolysaccharide-induced RAW264.7 cells. CA significantly decreased the bacterial burden in the lungs, encouraged the phagocytosis of alveolar macrophages, decreased the lung inflammatory injury of CLP-induced ARDS animals, and increased the survival rate [19]. Furthermore, investigations have indicated that CA can enhance the expression of the Bax and casp3 genes, as well as the p38 MAPK and JUN genes. Furthermore, limiting the circulation and migration of tumor cells in A549 cells, as well as blocking the production of the anti-apoptosis genes, cellular inhibitor of apoptosis protein 1 (cIAP1) and cIAP2, in the downstream NF-κB signaling pathway has anti-cancer effects. LBP has been linked to anti-inflammatory, antioxidant, anti-aging, and hypoglycemic effects through signaling pathways including NF-κB, PI3K-Akt-mTOR, p38-MAPK, Wnt-β-catenin, PI3K-Akt-eNOS, MLCK-MLC, and MyD88 [47]. The toxicology of healthy lungs is greatly influenced by alveolar macrophages. When LPS stimulates macrophages, they release a variety of substances, including cytokines and chemokines, which attract inflammatory cells to the lungs and cause inflammation. These lung disorders’ early stages are linked to inflammation. Thus, utilizing the rat alveolar macrophage NR8383 as a model, we examined the protective impact of a chlorogenic acid and LBP complex on LPS-induced inflammation in the current study.

This study aimed to investigate the in vitro antioxidant properties of the CA-LBP complex, which consists of chlorogenic acid and LBP. The experiment used four different ratios of chlorogenic acid to LBP (1:3, 1:5, 1:7, and 1:9) to determine the optimal ratio that can scavenge free radicals in ABTS. The results showed that the ratio of 1:7 had the highest scavenging capacity for ABTS, DPPH, and hydroxyl radicals. The Combination Index (CI) was used to measure the synergistic or antagonistic effects between the ratios, and the CA-LBP complex had a synergistic antioxidant effect within a certain concentration range. The experiment also aimed to investigate the anti-inflammatory effects of the CA-LBP complex. Previous studies showed that the phenolic compounds in chrysanthemum and polysaccharides in LBP can participate in the MAPK, PI3K/Akt, and NF-κB pathways, which can exert anti-inflammatory effects [26]. In this experiment, a model of LPS-induced inflammation in rat alveolar macrophages was constructed to test the effect of the single component and the complex on the release of NO and ROS. The results showed that the CA-LBP complex significantly inhibited LPS-induced NO production and effectively reduced the LPS-induced release of ROS, with cellular antioxidant activity. Important pro-inflammatory cytokines that orchestrate both local and systemic inflammation following damage and contribute to apoptosis are TNF-α, IL-6, and IL-1β. TNF-α, IL-6, and IL-1β levels were significantly elevated in the model group’s cells following LPS treatment in the current investigation, which is in line with previous work [48]. Furthermore, the complex was found to inhibit TNF-α, IL-6, and IL-1β content to alleviate inflammatory injury. However, the specific mechanism needs further exploration and investigation, particularly as to the role of the intracellular NF-κB signaling pathway in alveolar macrophages.

## 5. Conclusions

This research has demonstrated that chlorogenic acid and *Lycium barbarum* polysaccharides exhibit synergistic antioxidant properties, as evidenced by in vitro antioxidant property experiments across various concentrations. The optimal synergistic ratio, as determined by the Combination Index (CI), was found to be CA: LBP = 1:7, which displayed the highest antioxidant activity among the tested combinations. The CA-LBP complex has demonstrated both antioxidant and anti-inflammatory properties. It lowers LPS-induced nitric oxide and reactive oxygen species generation, as well as suppressing the release of inflammatory cytokines like TNF-α and IL-6 in NR8383 macrophages. As a result, it helps to reduce cellular inflammatory injury.

The interaction between polyphenols and polysaccharides is important in food processing. Bio-nanocomposites made of polyphenols and polysaccharides, for instance, can be utilized as protective coverings for fruit preservation in food preservation, lowering oxidative degradation and microbiological growth. In the medical field, polysaccharide lipid nanocarriers supplemented with curcumin can improve the efficacy of oral administration related to cellular lung cancer, and have the advantages of a simple operation, low cost, and high safety. Oral curcumin–polysaccharide lipid nanocarriers can overcome gastrointestinal disorders, improve the absorption effect, and further improve drug bioavailability and therapeutic effects. In addition, complexes formed by physically or chemically linking polysaccharides and polyphenols can be used for the development of food products targeting populations with high-risk health problems, e.g., type II diabetes, obesity, and cardiovascular diseases. Therefore, the compound shows great potential in the development of functional food and can be widely used in the food industry, healthcare, biofilm materials, and many other fields [31,49].

## Figures and Tables

**Table 1:** Monosaccharide content of *Lycium barbarum* polysaccharide samples.

	Mannose	Rhamnose	Galacturonic Acid	Glucose	Galactose	Arabinose
Content (mg/g)	1.97 ± 0.05	17.92 ± 0.67	2.91 ± 0.14	38.78 ± 1.61	383.57 ± 5.21	3.93 ± 0.15

Note: every experiment was carried out in triplicate, and the means of the same line with various lowercase characters differ noticeably. (*p* < 0.05). *n* = 3.

**Table 2:** Antioxidant activity of CA at different concentrations in vitro.

Concentration of CA (μg/mL)	ABTS (%)	DPPH (%)	Hydroxide Radical (%)
20	16.79 ± 3.73 ^g^	17.18 ± 0.35 ^g^	10.24 ± 1.26 ^g^
40	28.96 ± 1.23 ^f^	31.55 ± 0.41 ^f^	16.85 ± 0.51 ^f^
60	41.89 ± 1.59 ^e^	44.78 ± 0.83 ^e^	22.38 ± 0.40 ^e^
80	55.06 ± 2.28 ^d^	55.48 ± 1.85 ^d^	30.21 ± 0.50 ^d^
100	67.78 ± 1.60 ^c^	65.87 ± 2.47 ^c^	35.49 ± 0.43 ^c^
120	81.44 ± 1.87 ^b^	74.69 ± 2.39 ^b^	40.48 ± 0.72 ^b^
140	88.08 ± 2.57 ^a^	84.81 ± 1.37 ^a^	46.25 ± 0.37 ^a^

Note: all experiments were repeated three times and the results with mean and standard values. Distinct letters for each column unit indicate significant differences (*p* < 0.05).

**Table 3:** Antioxidant properties of LBP at different concentrations in vitro.

Concentration of LBP (mg/mL)	ABTS (%)	DPPH (%)	Hydroxide Radical (%)
1	19.26 ± 0.62 ^g^	21.82 ± 1.90 ^g^	44.68 ± 0.34 ^f^
2	36.31 ± 0.95 ^f^	32.55 ± 1.69 ^f^	51.41 ± 1.53 ^e^
3	53.95 ± 1.19 ^e^	40.28 ± 0.71 ^e^	57.65 ± 0.16 ^d^
4	69.01 ± 0.68 ^d^	48.10 ± 1.05 ^d^	60.30 ± 0.11 ^c^
5	79.65 ± 0.57 ^c^	54.75 ± 1.47 ^c^	62.28 ± 1.27 ^cd^
6	87.29 ± 0.21 ^b^	62.08 ± 0.07 ^b^	64.92 ± 0.51 ^b^
7	90.28 ± 0.05 ^a^	66.39 ± 0.74 ^a^	67.21 ± 0.46 ^a^

Note: all experiments were repeated three times and the results with mean and standard values. Distinct letters for each column unit indicate significant differences (*p* < 0.05).

## Data Availability

The original contributions presented in the study are included in the article, further inquiries can be directed to the corresponding author.
